# Comparative Study of Hearing Impairment among Healthy and Intensive Care unit Neonates in Mashhad, North East Iran

**Published:** 2015-07

**Authors:** Ahmadshah Farhat, Mohammad Mehdi Ghasemi, Javad Akhondian, Ashraf Mohammadzadeh, Habibollah Esmaeili, Rana Amiri, Ali Asqar Raoof Saeb, Mohammad Reza Tale, Faezeh Madani Sani

**Affiliations:** 1 *Neonatal Research Center, Imam Reza Hospital, Faculty of Medicine, Mashhad University of Medical Sciences, Mashhad, Iran. *; 2 *Sinus and Surgical Endoscopic Research Center,* *Faculty of Medicine, Mashhad* *University of Medical Sciences, Mashhad, Iran.*; 3 *Department of Pediatrics, Ghaem Hospital, Mashhad University of Medical Sciences, Mashhad, Iran.*; 4 *Audiologist, Member of Khorasan Cochlear Implant, Mashhad, Iran.*; 5 *Medical Student(Intern), Department of Pediatrics, Imam Reza Hospital, Mashhad University of Medical Sciences, Mashhad, Iran.*

**Keywords:** Hearing Impairment, Oto Acoustic Emission, Neonatal Intensive Care Unit, Rooming-in care.

## Abstract

**Introduction:**

According to World Health Organization (WHO) 2001 statistics, hearing disorders are the most common congenital disease, and the incidence rate among high-risk newborns is as much as ten times as high as that in healthy neonates. However, 78% of screening test failures are well-baby nursery babies. The Joint Committee on Infants’ Hearing (JCIH) has emphasized the importance of early diagnosis and treatment in neonates with hearing impairments in order to preserve their maximum linguistic skills. The aim of our study was to compare the prevalence of hearing loss among babies in the neonatal intensive care unit (NICU) and the rooming-in unit (RIU).

**Materials and Methods:**

Neonates born in three hospitals in Mashhad between 2008 to 2010 were studied prospectively and screened for auditory disorders using the oto acoustic emission (OAE) test at the time of discharge and 3 weeks later. To confirm hearing loss, the auditory steady state response (ASSR) test was used among those participants who failed both OAE tests.

**Results:**

Two-thousand and sixty-three neonates from the NICU were screened and compared with a control group consisting of 8,724 neonates from the RIU or the well-baby nursery. At the end of the study, hearing impairment as confirmed by failure in the ASSR test was diagnosed in 31 neonates (26 in the control group [0.30%] and five in the NICU group [1.94%]).

**Conclusion:**

In our study, the prevalence of hearing disorders among NICU neonates was 6.5-times greater than that among babies from the RIU or well-baby unit. This observation demonstrates the importance of universal screening programs particularly for high-risk population neonates.

## Introduction

According to World Health Organization (WHO) statistics, nearly 250 million persons were affected by hearing impairment of some description in 2001, with auditory disorders being the most common congenital disease (1-5). It has been demonstrated that the incidence rate of auditory disorders among high-risk newborns is up to 10 times higher than that in healthy neonates (6). However, 78% of failure cases in primary screening tests are among well-baby nursery neonates (7). The Joint Committee on Infants’ Hearing (JCIH) has emphasized the importance of the early diagnosis and treatment of neonates with hearing impairments. The goal of early hearing detection and intervention (EHDI) is to preserve the maximum linguistic acquisition skills. JCIH strongly recommends that all neonates should be screened for auditory impairments up to 1 month of age, and if any problems are detected in primary tests, a comprehensive hearing assessment should be performed before 3 months of age. In those with confirmed hearing impairments, therapeutic intervention should be performed up to 6 months of age (8). Researchers have demonstrated that the final outcome in infants who receive therapeutic intervention below 6 months of age is much higher than among those who are treated later, and that negligence in this regard may lead to verbal developmental delay (9-11). 

Different studies have reported various risk factors for hearing loss (8,12). However, it has been noted that half of the patients with confirmed hearing impairment have no known JCIH risk factors. Therefore, screening limited to a high-risk population may lead to numerous patients being missed (13,14). Otoacoustic emission (OAE) and auditory steady state response (ASSR) tests are the most common tests in neonatal wards; with the ASSR test being utilized in case of false referral from the OAE test (15-17). JCIH recommends that at least one auditory brainstem response (ABR) test be performed as a confirmatory audiology diagnostic evaluation in young children, while researchers have demonstrated that the ASSR test is a reasonable alternative to ABR and can be used to determine the pure tone threshold in neonates at risk for auditory disorders (8,18-19). 

The aim of our study was to compare the prevalence of hearing loss in neonatal intensive care unit babies (high-risk group) with well-baby nursery babies (control group) using OAE and ASSR tests.

## Materials and Methods

This prospective study was conducted from November 2008 to November 2010 in three university-related hospitals in Mashhad, Northeast Iran. Neonates born in Imam Reza, Qaem or Ommol Banin Hospitals were screened for auditory disorders in order to evaluate hearing impairments.All neonates before discharge were screened, whether they were in the neonatal intensive care unit (NICU) (case group) or in the well-baby rooming-in unit (RIU) (control group). The OAE test (Ero-Scan model, Maico USA company) was used as the first step in the screening program. Neonates who failed in the primary test were followed-up with a second OAE test after a 3-week gap. Failure in the secondary test led to hearing loss being confirmed in the ASSR test (Eclipse EP-25 model, Interacoustic Denmark). The frequency of hearing impairment was assessed, and data analysis was performed using SPSS software.

## Results

A total of 263 neonates from the NICU participated in this study (case group). Overall, 185 cases (70.3%) failed in first OAE test, of whom 180 neonates (97.3%) participated in the secondary OAE test to confirm their first test results. One hundred cases (38.8% of the total study neonates) were referred again after the second OAE test. 

Ninety-nine neonates (99% of those who failed the second test or 37.6% of the original 263 neonates) underwent the ASSR test. Unilateral or bilateral hearing impairment was confirmed in five neonates who failed the 25 db in four 500, 1000, 2000,4000 hearing thresholds in ASSR test. In other words, 1.9% of 263 NICU participants had a hearing impairment in the case group.

In the control group 8,724 well newborns from the RIU participated; of whom 1,046 cases failed in the first OAE test (12.0%). Eight-hundred and twenty-six neonates (79.0% of those who failed the first OAE test) participated in a second OAE test, which was failed by 419 neonates (4.9% of the case group; Table 1). Finally 26 of 8,726 neonates in the control group (0.3%) demonstrated unilateral or bilateral neurosensorial hearing impairment in the ASSR test. This rate is 6.64-times lower than the NICU cases in the case group.

**Table1:** Frequency of hearing impairment in case and control groups in consecutive steps of study

	Participated in first OAE Test N(P)	Failed in First OAE Test N (%)	Participated in second OAE Test N (% who failed first OAE)	Failed in second OAE Test N (%Total Part.)	Participated in ASSR Test N (%who failed second OAE))	Failed in ASSR N (% Total part.)
RIU	8724 (100)	1046 (11.98)	826 (78.96)	419 (4.92)	417 (99.52)	26 (0.30)
NICU	263 (100)	185 (70.34)	180 (97.29)	100 (38.75)	99(99)	5 (1.94)

## Discussion

OAE and ASSR tests are auditory tests commonly applied in neonatal wards, with the OAE test being the most utilized because of its ready availability. In our study, we used the OAE test as the primary screening test and applied the ASSR test for confirmation of hearing impairment. 

The study showed that there were false positive cases in both the case and control groups, but the rate of false positives was higher in the case group than in the control group. The rate of false referrals from the OAE test has been estimated at approximately 7% (±3%) (15-17). In our study, the neonatal hearing impairment was approximately 3.5 neonates per 1,000 live births. According to universal statistics, this rate has been reported at approximately two to five neonates in 1,000 live births (6). In a 2007 study by Korres et al., it was demonstrated that 78% of positive screening tests are from healthy babies with no risk factors (7). In our survey, 83% of positive screening tests were from well babies. Additionally, the JCIH 2007 guidelines state that half of all patients with confirmed hearing loss have no known risk factors for auditory disorders, emphasizing the value of universal screening programs rather than selective high-risk population screening (8). It is known that the prevalence of hearing impairments in high-risk neonates could be up to 10-times higher than that in well babies (6).

In a 2004 study conducted in Tehran University, 16% of neonates with NICU hospitalization had hearing loss. Although the authors demonstrated no significant relationship between NICU admission and hearing loss (20), a study by Ur Rehman et al. in 2012 reported that 1.7% of NICU neonates had a hearing deficit compared with 0.2% of healthy newborns (21). In 2010, researchers in the Netherlands reported a 1.8% prevalence rate for hearing impairment among NICU neonates. They studied different risk indicators and their effect on variations in the prevalence of hearing loss in NICUs in the Netherlands, and found ethnicity to be a risk indicator (22). In another study in the Netherlands, the prevalence of hearing loss among NICU newborns was cited as 3.2% (23). In 1999, an evaluation of the efficacy of universal newborn hearing screening reported an estimated incidence of hearing deficit in NICU neonates of 2–4% (17). In our study, the prevalence of hearing disorders among NICU neonates was 1.9% (five out of 263), compared with 0.3% in RIU babies. This indicates a 6.5-times higher rate of hearing impairment in NICU neonates known as high-risk babies for auditory disorders. 

## Conclusion

In our study, the rate of hearing disorders among NICU neonates was 1.9%; 6.5-times that of RIU babies. In total, 84% of failure cases in screening tests were from RIU babies. This indicates that universal screening programs should be preferred over selective high-risk population screening.
